# The complete chloroplast genome sequence of red raspberry (*Rubus idaeus* L.) and phylogenetic analysis

**DOI:** 10.1080/23802359.2024.2397986

**Published:** 2024-08-30

**Authors:** Hao Zhou, Huajie Zhang

**Affiliations:** a Key Laboratory of Molecular Biophysics of the Ministry of Education, College of Life Science and Technology, Huazhong University of Science and Technology, Wuhan, China; b CAS Key Laboratory of Plant Germplasm Enhancement and Specialty Agriculture, Wuhan Botanical Garden, Chinese Academy of Sciences, Wuhan, Hubei, China; c Center of Conservation Biology, Core Botanical Gardens, Chinese Academy of Sciences, Wuhan, Hubei, China; d Wuhan Botanical Garden, University of Chinese Academy of Sciences, Beijing, China

**Keywords:** Phylogeny, plastome, Rosaceae, *Rubus idaeus*

## Abstract

Red raspberries, *Rubus idaeus* L. 1753 are famous fruits which possess high value bioactive compounds. In this study, we report the complete chloroplast genome of *R. idaeus*, it displayed a typical quadripartite structure with 155687 bp in length. The genome encodes 127 genes including 79 protein coding genes, 8 rRNA genes and 40 tRNA genes, the overall GC content is 37.2%. Phylogenetic analysis revealed a close relationship between *R. idaeus* and *R. sachalinensis* in Section Malaehobatus.

## Introduction

**Figure 1.**
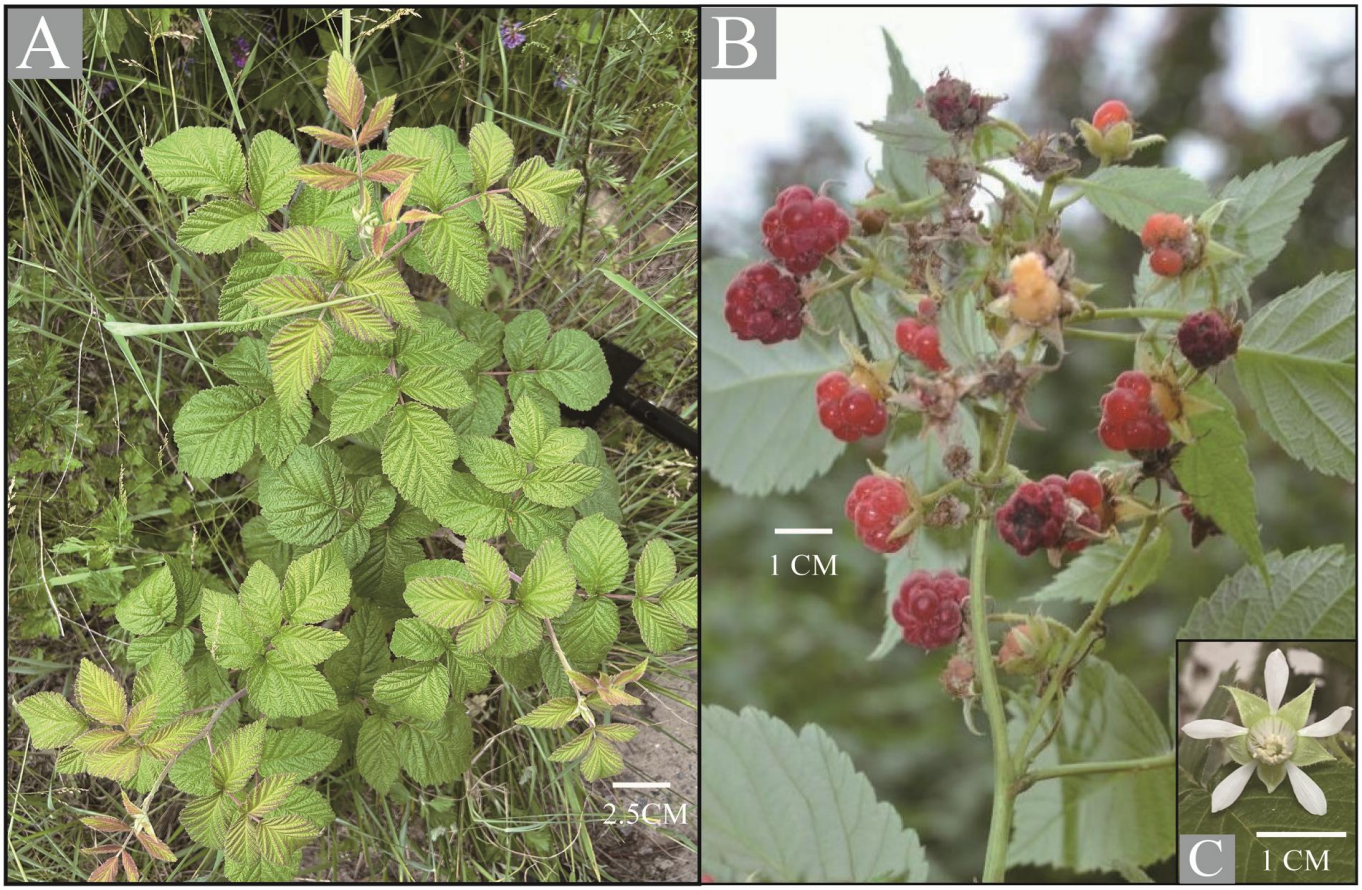
The photo of the whole plant *Rubus idaeus*. (A) The whole plant. (B) The fruits. (C) The flower. Morphology and habitat map of *R. idaeus* from Heicha Mountain, Xihui Village, Xing Town, Lvliang City, Shanxi Province, China (38°23′48″ N; 111°27′44″ E), photograph by Huajie Zhang.

Red raspberries, *Rubus idaeus* L. 1753 are perennial shrubs 1–2 m tall in Rosaceae, it is native fruit from Europe (Figure 1) (Ispiryan et al. 2021; Lopez-Corona et al. 2022). As an important commercial fruit crop, red raspberries are widely cultivated in all temperate regions of the world now (Ispiryan et al. 2021; Davik et al. 2022). This berry has been shown to be rich in anthocyanins and other phenolic compounds with a strong antioxidant capacity (Kähkönen et al. 2001; Kafkas et al. 2008; Çekiç and Özgen, 2010). It also comprises rich health-beneficial nutrients such as minerals, vitamins and organic acids (Beekwilder et al. 2005; Pantelidis et al. 2007). The raspberry seed oil displays anti-inflammatory activity, which can be used to prevent gingivitis and skin lesions and is therefore added to products such as sunscreen and toothpaste (Oomah et al. 2000; Ispiryan et al. 2021; Lopez-Corona et al. 2022).

*Rubus* is a large genus in Rosaceae which contains plentiful species with similar morphologies, causing complicated arguments on the species delimitation (Wang et al. 2016; Carter et al. 2019). Molecular identification is an efficient approach for species-level differentiation *via* standard DNA barcodes. Up to now, the complete plastid sequences of many *Rubus* species have been assembled and reported to construct the phylogeny of *Rubus* (Yu et al. 2022). The chromosome-level genome sequence of red raspberries was also detected (Davik et al. 2022). However, no complete chloroplast genome research has been conducted for the important fruit crop red raspberries. Here, we sequenced and assembled the complete chloroplast genome of wild accessions of red raspberries *R. idaeus*, it can not only provide genomic molecular maker to clarify the phylogenetic position of this species within *Rubus*, but also can help to explore the genetic diversity and promote the breeding programs.

## Materials and methods

The sample of *Rubus idaeus* was collected from Heicha Mountain in northwestern Shanxi Province (38°23′48″ N; 111°27′44″ E). The specimen was deposited in Wuhan botanical Garden with the specific identifying number of HSM-2023fu (contacts: Guangwan Hu, guangwanhu@wbgcas.cn). We collected fresh leaves in silica gel to extract total genomic DNA. 1.5 µg of DNA of sample was sequenced using illumine Hiseq Platform, a minimum 5GB of raw sequencing data was finally obtained. The chloroplast genome of *R. idaeus* was assembled with NOVOPlasty (Dierckxsens et al. 2017), the read coverage depths of the assembled genome can be seen in Supplementary Figure 1. Then we annotated the genome with Goseq (https://chlorobox.mpimp‐golm.mpg.de/geseq.html) and manually checked and modified the start/stop codons of genes in Geneious v9.0.2 (Kearse et al. 2012). The complete chloroplast genome of *R. idaeus* was submitted to Genbank with the accession number of OR698909. Simple Sequence Repeats (SSRs) were detected in MISA with default parameters (Beier et al. 2017).

The complete genome sequences of *R. idaeus* and other 39 representative species from Rosaceae family were downloaded from NCBI, two species in *Rosa* were selected as outgroups (Figure 3). We extracted the sequences of large single copy (LSC) region, small single copy (SSC) region and one repeat of inverted repeated (IR) region of each species separately, then concatenated sequences into a super-matrix in Geneious v9.0.2 (Kearse et al. 2012). Concatenated sequences were then aligned with MAFFT v7.5(Katoh and Standley 2013). We conducted the maximum-likelihood (ML) analysis with RAxML v8.2.12 (Stamatakis 2014) with the model GTRGAMMA, 1000 rapid bootstrap replicates were conducted. We also detected the nucleotide diversity (pi) of the super-matrix in DnaSP 6 (Rozas et al. 2017) with a step size 400 bp and a window length 600 bp.

## Results

**Figure 2.**
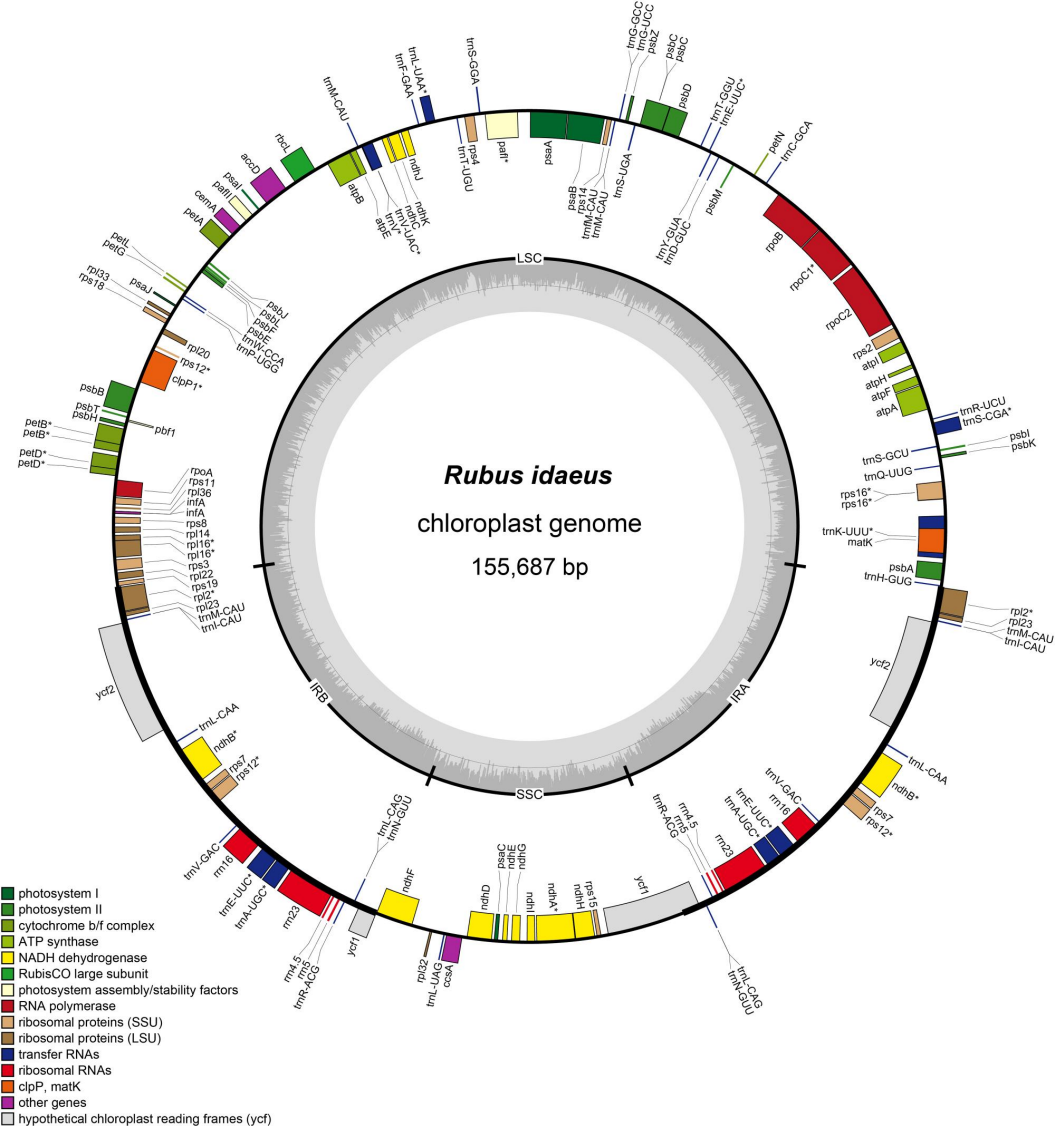
Circular maps of the *Rubus idaeus* chloroplast genome. Genes shown inside the circle are transcribed clockwise, and those outside the circle are counterclockwise transcribed. The light grey and the darker grey in the inner circle correspond to at and GC content, respectively. Different functional groups are signed according to the colored legend. LSC: large single copy, SSC: small single copy; IRA/IRB: Inverted repeat regions a/B.

**Figure 3.**
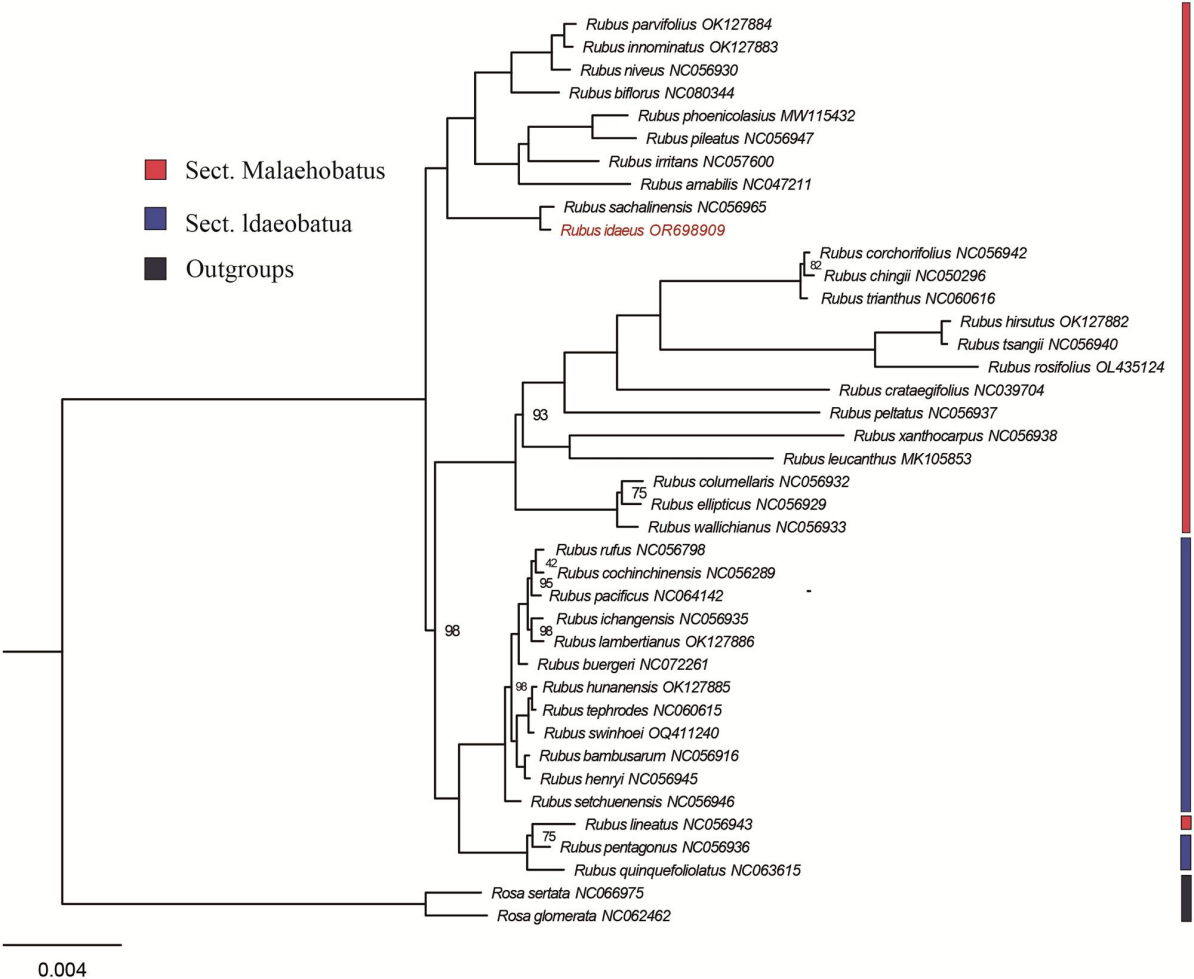
Chloroplast phylogeny of 38 *Rubus* species using maximum likelihood (ML) method based on the concatenated LSC, SSC and an IR region. The red fonts represent *R. idaeus* in this study. Two *Rosa* species (*Rosa sertata* and *Rosa glomerata*) were selected as outgroups. Genbank IDs were listed after the species’ names. Maximum likelihood bootstrap values (BS) are shown at nodes. Branches with no values listed have 100% BS. The following sequences were used: *Rubus parvifolius* OK127884 (Yu et al. 2022), *Rubus innominatus* OK127883 (Yu et al. 2022), *Rubus niveus* NC056930, *Rubus biflorus* NC080344, *Rubus phoenicolasius* MW115432 (Zhang et al. 2021), *Rubus pileatus* NC056947, *Rubus irritans* NC057600 (Han et al. 2023), *Rubus amabilis* NC047211, *Rubus sachalinensis* NC056965 (Liu et al. 2021), *Rubus idaeus* OR698909 (this study), *Rubus corchorifolius* NC056942, *Rubus chingii* NC050296 (Wang et al. 2020), *Rubus trianthus* NC060616 (Yu et al. 2022), *Rubus hirsutus* OK127882 (Yu et al. 2022), *Rubus tsangii* NC056940, *Rubus rosifolius* OL435124 (Yang et al. 2022), *Rubus crataegifolius* NC039704 (Yang et al. 2017), *Rubus peltatus* NC056937, *Rubus xanthocarpus* NC056938, *Rubus leucanthus* MK105853, *Rubus columellaris* NC056932, *Rubus ellipticus* NC056929, *Rubus wallichianus* NC056933, *Rubus rufus* NC056798, *Rubus cochinchinensis* NC056289, *Rubus pacificus* NC064142 (Xiong et al. 2022), *Rubus ichangensis* NC056935, *Rubus lambertianus* OK127886 (Yu et al. 2022), *Rubus buergeri* NC072261, *Rubus hunanensis* OK127885 (Yu et al. 2022), *Rubus tephrodes* NC060615 (Yu et al. 2022), *Rubus swinhoei* OQ411240, *Rubus bambusarum* NC056916, *Rubus henryi* NC056945, *Rubus setchuenensis* NC056946, *Rubus lineatus* NC056943, *Rubus pentagonus* NC056936, *Rubus quinquefoliolatus* NC063615, *Rosa sertata* NC066975, *Rosa glomerata* NC062462 (Chen et al. 2022).

The length of the complete chloroplast genome of *R. idaeus* was 155687 bp, with an LSC region of 85038 bp, an SSC region of 18717 bp and two separated IR regions of 25966 bp (Figure 2). A total of 127 genes were identified in the cp of *R. idaeus*, including 79 protein-coding genes, 40 tRNA genes, and 8 rRNA genes. Twelve genes contain introns, *rps1*2 is a trans-splicing gene (Supplementary Figures 2 & 3). The overall GC content was 37.2%, and the corresponding contents for LSC, SSC and IR regions were 35.2%, 31.3% and 42.8%, respectively. The genome included 20 duplicated genes in the IR region including *ycf*1, *trn*N-GUU, *trn*L-CAG, *trn*R-ACG, *rrn*5, *rrn*4.5, *rrn*23, *trn*A-UGC, *trn*E-UUC, *rrn*16, *trn*V-GAC, *rps*12, *rps*7, *ndh*B, *trn*L-CAA, *ycf*2, *trn*I_CAU, *trn*M-CAU, *rpl*23 and *rpl*2, which exhibited 50.6% protein-coding sequences. Moreover, a total of 46 SSRs were identified in the chloroplast genome of *R. idaeus*. The phylogenetic results revealed that both Sect. Malaehobatus and Sect. Idaeobatua are polyphyletic groups, which is corresponding with previous research (Wang et al. 2016). Phylogenetic analysis also demonstrated a close relationship between *R. idaeus* and *R. sachalinensis* with 100% bootstrap support value (Figure 3). The nucleotide diversity analysis revealed four highly divergent intergenic regions (pi > 0.03), including *rps*16*-trn*Q, *trn*T*-trn*L, *pet*A*-psb*L and r*pl*32*-trn*L (Supplementary Figure 4).

## Discussion and conclusion

Raspberry has important medicinal and therapeutic value, it can be used in food, pharmaceutical, cosmetic and chemical industries. In this study, we successfully sequenced and assembled the complete chloroplast genome of *R. idaeus* and constructed the phylogenetic tree of *Rubus*, revealed that Sect. Malaehobatus and Sect. Idaeobatua are polyphyletic clades, and clarified the close relationships between *R. idaeus* and *R. sachalinensis.* The phylogenetic tree is consistent with previous research (Wang et al. 2016) with higher bootstrap support values, it shows that chloroplast genome sequence is an effective tool to construct the phylogenetic relationships of Rosaceae. The SSRs and the nucleotide diversity analysis can help to detect the population structure of wild populations. Four highly divergent intergenic regions can be used for phylogeny and evolution analysis in future studies. The first complete chloroplast genome will be beneficial to the identification and development of germplasm resources of raspberry in further research.

## Supplementary Material

The supplemental figures.docx

Figure S2.jpg

Figure S3.jpg

Figure S1.jpeg

## Data Availability

The genome sequence data supporting the funding is available in the GenBank of NCBI at https://www.ncbi.nlm.nih.gov/ under accession number OR698909. The associated BioProject, SRA and Bio-Sample numbers are PRJNA1033369, SRR26558505 and SAMN38031542, respectively.
