# Supplementary material for: The complete chloroplast genome sequence of red raspberry (Rubus idaeus L.) and phylogenetic analysis
Source: Mitochondrial DNA B Resour. 2024 Aug 30;9(9):1152–6. doi: 10.1080/23802359.2024.2397986 (PMC11370666; doi:10.1080/23802359.2024.2397986)
Supplement: The supplemental figures.docx [file TMDN_A_2397986_SM2026.docx]

**Supplemental material**

**Figure S1**. The sequencing depth of coverage plots of *Rubus idaeus*. The Illumina sequences were mapped to the chloroplast genome of *R. idaeus* in Geneious. We exported the coverage information and draw the line chart. The horizontal axis represents the length of genome, while the vertical axis represents the coverage depth.

**Figure S2** Schematic map of cis-splicing genes of *R. idaeus*. Genes were arranged based on the order in chloroplast genome. Gene names were listed on the left, the structures were displayed on the right. Exons were shown in black, intros were shown in white. The arrows indicated the directions of genes, the length of exons and introns were not drawn to scale.

**Figure S3** Schematic map of trans-splicing gene *rps12* of *R. idaeus*. The gene comprises three unique exons, two of them in the IR regions are duplicated.

**Figure S4** Nucleotide diversity (pi) of complete chloroplast genome sequence that removes one repeat of IR region among *Rubus*.
