# Supplementary figures and images for: The complete chloroplast genome sequence of red raspberry (Rubus idaeus L.) and phylogenetic analysis
Source: Mitochondrial DNA B Resour. 2024 Aug 30;9(9):1152–6. doi: 10.1080/23802359.2024.2397986 (PMC11370666; doi:10.1080/23802359.2024.2397986)

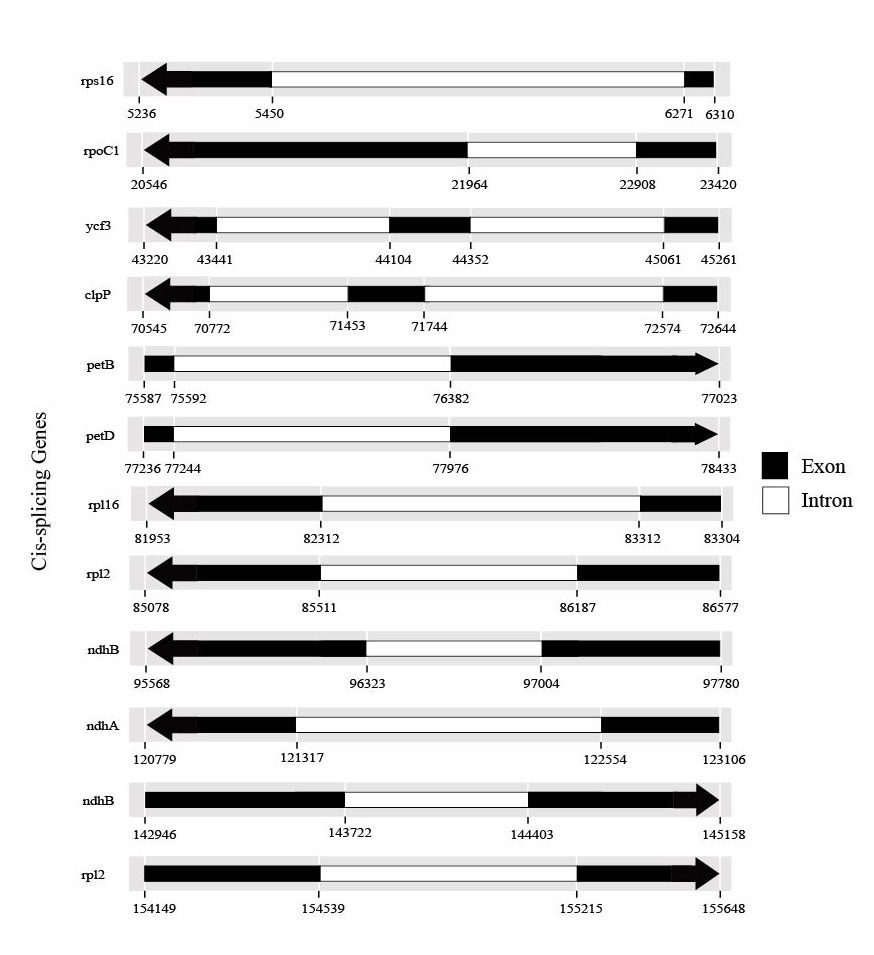

Supplement: Figure S2.jpg [file TMDN_A_2397986_SM2025.jpg]

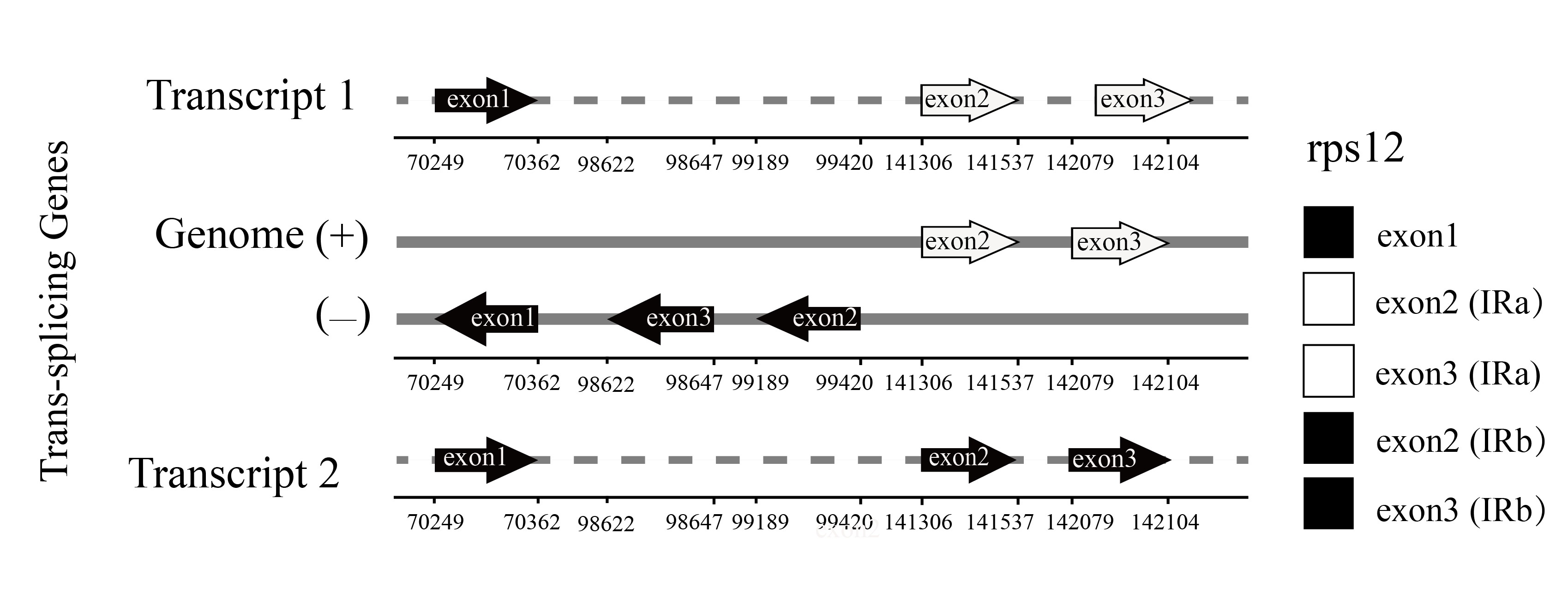

Supplement: Figure S3.jpg [file TMDN_A_2397986_SM2024.jpg]

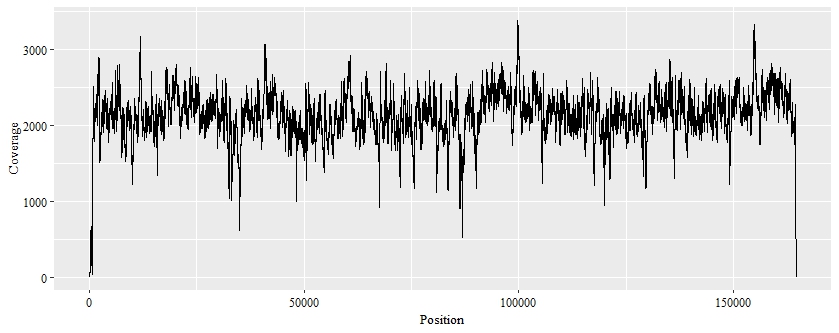

Supplement: Figure S1.jpeg [file TMDN_A_2397986_SM2023.jpeg]
